# Data on peptides identified by mass spectrometry analysis of in vitro DYRK1A-mediated phosphorylation sites on GLI1

**DOI:** 10.1016/j.dib.2017.09.057

**Published:** 2017-10-02

**Authors:** Ben K. Ehe, David R. Lamson, Michael Tarpley, Rob U. Onyenwoke, Lee M. Graves, Kevin P. Williams

**Affiliations:** a Biomanufacturing Research Institute and Technology Enterprise, North Carolina Central University, Durham, NC 27707, USA; b Department of Pharmaceutical Sciences, North Carolina Central University, Durham, NC 27707, USA; c Department of Pharmacology, School of Medicine, University of North Carolina, Chapel Hill, NC 27599, USA

## Abstract

The data presented in this article support the accompanying research article “Identification of a DYRK1A-mediated phosphorylation site within the nuclear localization sequence of the hedgehog transcription factor GLI1” (Ehe et al., 2017) [1]. Although it has been demonstrated that DYRK1A (dual-specificity tyrosine-regulated kinase 1A) can phosphorylate the hedgehog pathway transcription factor GLI1 (GLIoma-associated oncogene homolog 1) and promote its nuclear localization, the DYRK1A-mediated sites of phosphorylation on GLI1 involved were not fully known. This article details the mass spectrometry methods and resulting dataset for the peptides identified from GLI1 when incubated with DYRK1A under varying conditions. The data include details of sequence coverage and all phospho-peptides identified.

**Specifications table**

**Table:** 

Subject area	*Biology*
More specific subject area	*Mass spectrometry analysis of phospho-peptide sites,*
Type of data	*Table, Figures*
How data was acquired	*Velos-Orbitrap mass spectrometer (Thermo-Scientific), and reversed phase nano-HPLC, nanoAcquity UPLC system (Waters)*
Data format	*Analyzed*
Experimental factors	*Human recombinant GLI1 expressed in HEK293 cells and purified by anti-FLAG affinity capture; GLI1 incubated with DYRK1A*
Experimental features	*Tryptic peptide generation, identification of phospho-sites by mass spectrometry*
Data source location	*Durham, NC, USA*
Data accessibility	*Data are within this article.*

**Value of the data**

• Use of high resolution mass spectrometry to identify phospho-peptides from GLI1 after DYRK1A incubation.
• Identification of novel direct DYRK1A site of phosphorylation on GLI1.
• Only data available for DYRK1A mediated phosphorylation of GLI1 within its NLS.

## 1 Data

The dataset presented here represents details of peptides that were identified by mass spectrometry from recombinant human GLI1 under varying conditions. We also present methodology for GLI1 expression and purification.

For GLI1 phospho-peptide analysis, two independent experiments were carried out. For study 1, the samples were GLI1 alone (sample 1), GLI1 + DYRK1A + ATP (sample 2) and GLI1 + DYRK1A – ATP (sample 3) (Table 1). Samples were incubated for 30 min at 30 °C, electrophoresed on SDS-PAGE, and the bands representing GLI1 were excised and the gel slices divided into two parts. For each sample, tryptic peptides were generated from both gel pieces for mass spectrometry analysis. The summary results and complete phospho-peptide analysis for the major site (Ser408) of DYRK1A-mediated phosphorylation identified in Ehe et al. [1] are provided (Table 1, Table 2).

**Table 1:** Summary of sequence coverage, number of peptides and PSMs for analysis of GLI1 tryptic peptides by mass spectrometry.

Sample name	Sample #	Run^a^	Coverage (%)^b^	# Peptides	PSM^c^
GLI1	1	1	57.73	114	386
GLI1	1	2	48.35	76	279
GLI1 + DYRK1A + ATP	2	1	74.39	144	720
GLI1 + DYRK1A + ATP	2	2	72.22	143	692
GLI1 + DYRK1A - ATP	3	1	77.69	161	762
GLI1 + DYRK1A - ATP	3	2	61.98	118	543

a Each gel slice was divided into 2 pieces, treated and analyzed separately.

b Coverage (%) refers to the % coverage of the entire GLI1 protein.

c PSMs are the number of spectra that matched peptides and is an approximate estimate of amount.

**Table 2:** Summary of GLI1 peptides identified with the highest number of PSMs.

1^a^	1	2	2	3	3		Sequence	Modifications
**1**^b^	**2**	**1**	**2**	**1**	**2**	**Sum**^c^		
		6	6			12	APsISTVEPK	S3(Phospho)
10	10	8	7	8	9	52	FLGGSQVsPSR	S8(Phospho)
		4	2			6	FLGGSQVSPsRAK	S10(Phospho)
			1	2	2	5	FLGGSQVsPSRAK	S8(Phospho)
3	2	3	5	3	1	17	GGGTsPTAASSLDR	S5(Phospho)
1		1	1	1		4	RSsSSSSISSAYTVSR	S3(Phospho)
0	3	7	9	6	9	34	GSSGHtPPPSGPPNmAVGNmSVLLR	T6(Phospho); M15(Oxidation); M20(Oxidation)
		1	2	1	1	5	TsPSSLVAFINSR	S2(Phospho)
		5	2			7	GPsPSFGVQPcGPHDSAR	S3(Phospho); C11(Carbamidomethyl)
1	2	3	2	2	1	11	REPEsVYETDcR	S5(Phospho); C11(Carbamidomethyl)

a Sample

b Run as in Table 1. Gel samples were subjected to tryptic digest and TiO_2_ enrichment. Data from study 1 (Fig. 2A in Ehe et al. [1]).

c Modified peptides identified with at least 4 PSMs are listed.

**Fig. 1:**
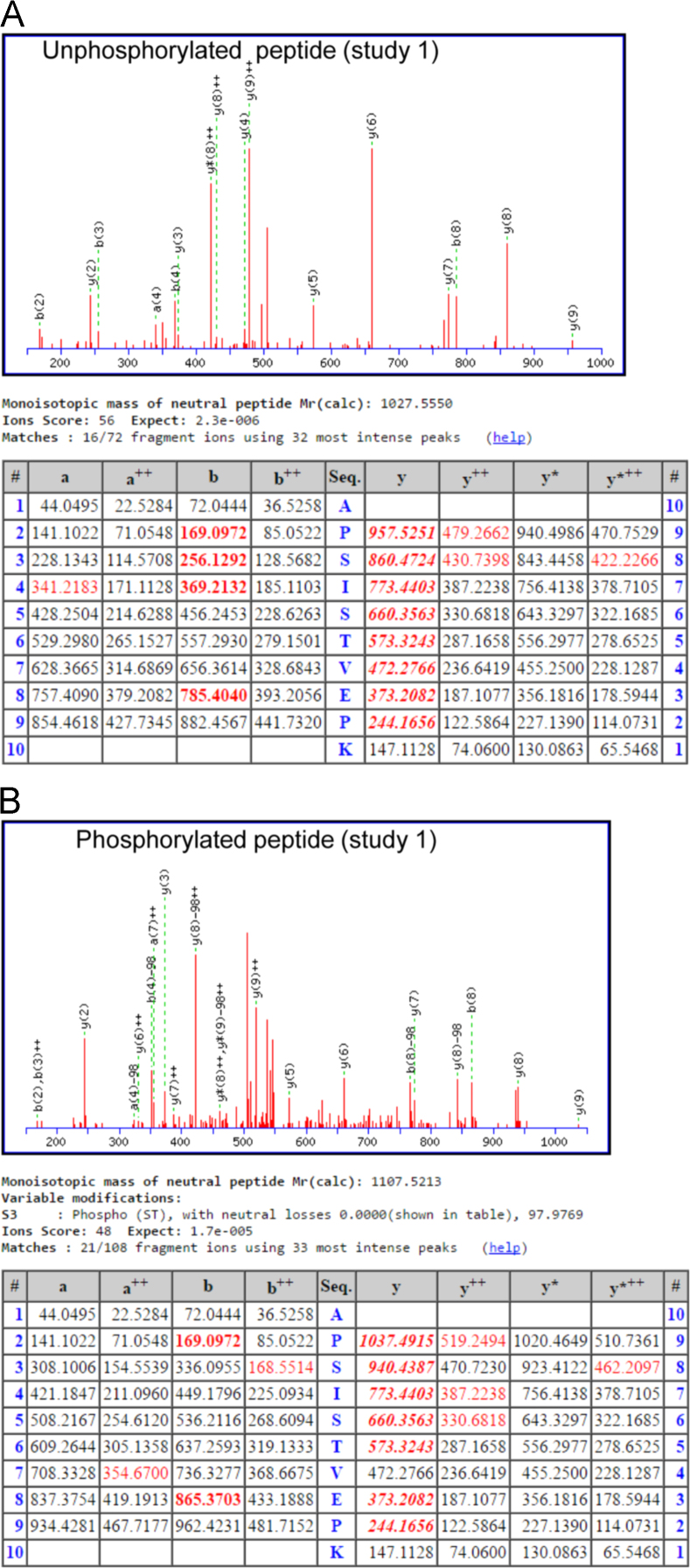
MS/MS fragmentation spectra and analysis for GLI1 tryptic peptide spanning Ser408. The MS/MS fragmentation spectra and Mascot results are shown for the non-phosphophorylated (A) and phosphorylated (B) APSISTVEPK peptide from study 1.

**Fig. 2:**
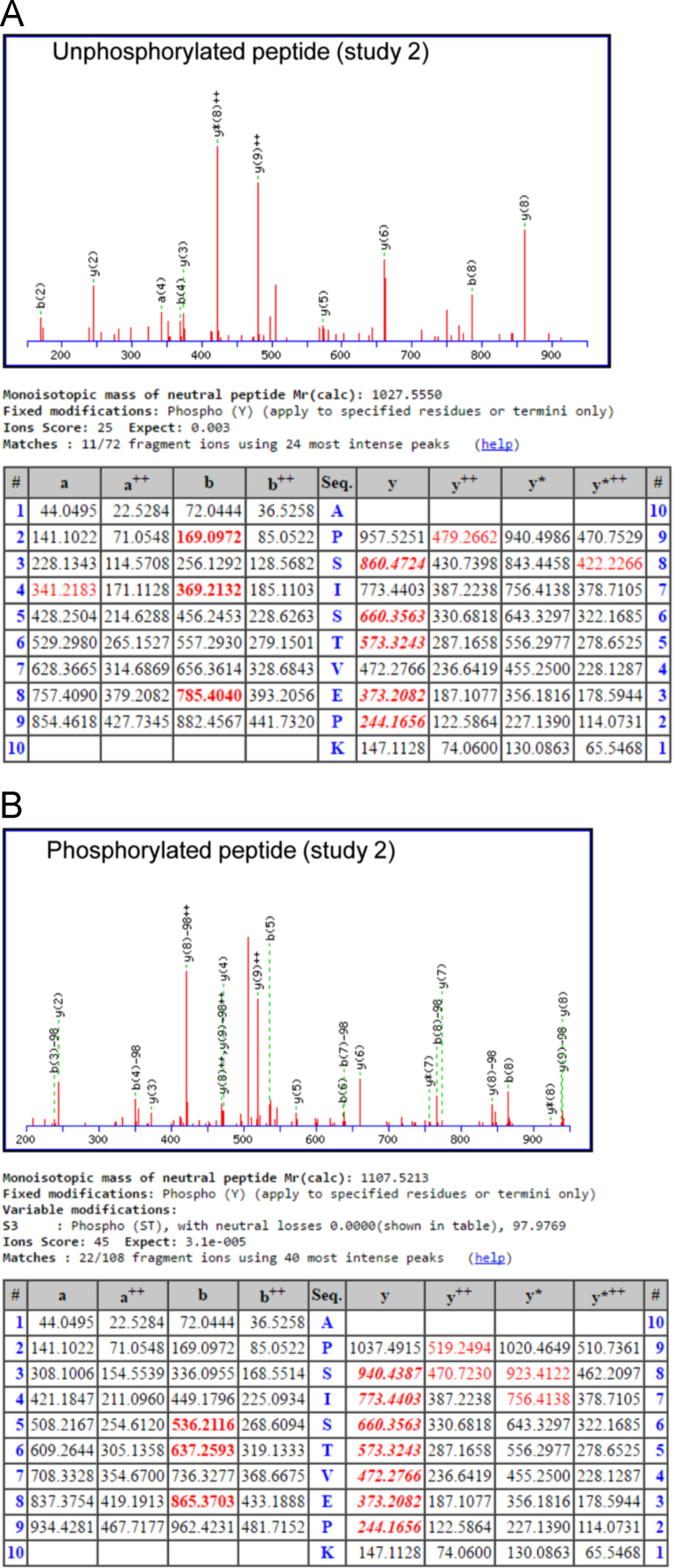
MS/MS fragmentation spectra and analysis for GLI1 tryptic peptide spanning Ser408. The MS/MS fragmentation spectra and Mascot results are shown for the non-phosphophorylated (A) and phosphorylated (B) APSISTVEPK peptide from study 2.

Table 1 summarizes for study 1 the % sequence coverage, # peptides and # PSMs identified from GLI1 when treated under the differing conditions. For each run of each sample, approximately 50–70% coverage, 100 peptides, and 279–720 peptide spectral matches (PSMs) were identified. Observed peptide masses were compared to the GLI1 protein sequence (NCBI reference sequence: NM_005269.2) using MASCOT software [2]. Those GLI1 peptides identified with the highest number of PSMs (≥ 4) are listed in Table 2. From these analyses, a number of high confidence phospho-peptides were identified with the majority present in all samples, suggesting those were related to basal GLI1 phosphorylation. The major difference between the different conditions was phosphorylation of the peptide APSISTVEPK at Serine 408 of GLI1. The unphosphorylated APSISTVEPK peptide was detected 10 to 13 times per run for each sample (Table 3). The phosphorylated peptide was detected 6 times in each of the analyses of sample 2 (GLI1 + DYRK1A + ATP) and not in any other run (Table 3). For study 2, the same conditions as study 1 were replicated plus an additional sample; the samples were GLI1 alone (sample 1), GLI1 + DYRK1A - ATP (sample 2) and GLI1 + DYRK1A + ATP (sample 3) and GLI1 + DYRK1A + ATP + harmine (sample 4). The major site of DYRK1A-mediated phosphorylation on GLI1 was again identified as Ser408, with 10 of 11 peptides (MH+ [Da] = 1108.52836790125) observed only in the +ATP, + DYRK1A sample (Table 4, see also Figs. 2A and 2B in Ehe et al. [1]). Fig. 1, Fig. 2 provide MS/MS fragmentation spectra and analysis for the unphosphorylated and phosphorylated APSISTVEPK peptide from studies 1 and 2, respectively.

**Table 3:** Summary of unphosphorylated and phosphorylated peptides detected for tryptic peptide spanning Ser408 of GLI1.

1^a^	1	2	2	3	3		Sequence	Modifications
**1**^b^	**2**	**1**	**2**	**1**	**2**	**Sum**		
13	13	12	10	11	12	72	APSISTVEPK	
		6	6			12	APsISTVEPK	S3(Phospho)
		1				1	APsIsTVEPK	S3(Phospho); S5(Phospho)

a Sample

b run as in Table 1.

**Table 4:** Summary of GLI1 peptides identified with modifications from study 2.

1^a^	1	2	2	3	3	4	4		Sequence	Modifications
**1**^b^	**2**	**1**	**2**	**1**	**2**	**1**	**2**	**Sum**^c^		
				8	2		1	11	APsISTVEPK	S3(Phospho)
2	1	2	1	2			1	9	FLGGSQVsPSR	S8(Phospho)
1	1	2		5	1		1	11	GGGTsPTAASSLDR	S5(Phospho)

a Sample.

b Run from study 2. Gel samples were subjected to tryptic digest and TiO_2_ enrichment. Data from experiment B (Fig. 2B in Ehe *et al*. [1]).

c Modified peptides identified with at least 4 PSMs are listed.

## 2 Experimental design, materials and methods

### 2.1 GLI1 protein expression and purification

Recombinant human GLI1 protein was produced by transfection of the pCMV6 GLI1-myc-DDK plasmid (Origene, Rockville, MD) into HEK-293 cells (CRL-1573; ATCC, Manassas, VA). Cells were grown in Eagle's Minimum Essential Medium supplemented with 10% FBS. HEK-293 cells were seeded in T150 culture flasks and allowed to reach 80–90% confluence. For transfection, a ratio of 1:3 (DNA to Turbofectin 8.0 transfection reagent (Origene)) was used. Turbofectin (45 µL) was first dissolved in OptiMEM serum-free medium and incubated for 5 min. DNA (15 µg) was then added and incubated for 30 min to allow DNA complex formation. The complex was added to the cells and after 24 h, media replaced and incubated for another 24 h.

For purification, cells were lysed in buffer supplemented with protease and phosphatase inhibitor cocktails. The cell extract was clarified by centrifugation at 14,000 rpm for 15 min. Anti-Flag affinity gel (100 µL) was transferred to a microcentrifuge tube and washed five times with TBS. The clarified cell extract was then added to the affinity gel and mixed by gentle inversion and incubated on a rocking platform for 2 h (4 °C). After binding, the sample was centrifuged at 3000 rpm for 1 min to pellet the resin and the supernatant removed. The affinity gel was washed with 10 volumes of TBS-T (TBS with 0.05% Tween 20). Elution was carried out using 0.1 M acetate (pH 3.0), and the elution fractions were neutralized with 1 M Tris buffer (pH 9).

### 2.2 In vitro kinase assay and gel slice isolation

Purified GLI1 protein (2 µg) was incubated with recombinant human DYRK1A protein (ThermoFisher Scientific; 1 µg) in kinase buffer (25 mM Tris–HCl (pH 7.5) + phosphatase inhibitors) with ATP (1 mM) for 30 min at 30 °C. Controls included no DYRK1A, no ATP and plus 1 µM harmine (a selective DYRK1A inhibitor [3]). SDS-PAGE sample buffer was added to each sample, and following heating at 95 °C for 5 min, samples were electrophoresed on a 4–12% Bis-Tris SDS-PAGE gel (Invitrogen; Carlsbad, CA) for 1 h at 120 V and bands detected with Coomassie blue staining. Gel bands were excised, reduced, alkylated, and digested with trypsin to generate tryptic peptides for mass spectrometry. Phospho-peptides were enriched using TiO_2_ beads.

### 2.3 Mass spectrometry analysis

Mass spectrometry was performed at the UNC-CH Proteomics Center (Chapel Hill, NC, USA). TiO_2_ beads were used to enrich for phosphopeptides. The tryptic peptides were then extracted, lyophilized, resuspended in 2% acetonitrile/98% (0.1% formic acid), and loaded onto a 2 cm long × 360 µm o.d. × 100 µm i.d. microcapillary fused silica pre-column packed with Magic 5 µm C18AQ resin (Bruker-Michrom Biosciences; Billerica, MA). After sample loading, the pre-column was washed with 95% solvent A (0.1% formic acid in water)/5% solvent B (0.1% formic acid in acetonitrile) for 20 min at a flow rate of 2 μL/min. The pre-column was then connected to a 360 µm o.d. × 75 µm i.d. analytical column packed with 22 cm of 5 µm C18 resin. The peptides were eluted (flow rate of 250 nL/min) by increasing the percentage of solvent B to 40% with a Nano-Acquity HPLC solvent delivery system (Waters Corp; Milford, MA). The LC system was directly connected through an electrospray ionization source interfaced to an LTQ Velos-Orbitrap ion trap mass spectrometer (Thermo Fisher Scientific; Waltham, MA), which determined the identity and phosphorylation status of the eluted peptides as previously described [4].

The mass spectrometer was controlled by Xcalibur software and operated in the data-dependent mode. Ions over the m/z range 400–2000 were recorded as the initial MS scans. The ten most abundant ions were automatically selected for collision-induced dissociation. All files were searched using MASCOT [2] (Matrix Science) using Proteome Discoverer against the protein of interest (GLI1, Origene ID RC201110). The search parameters included peptide mass tolerance of 10 ppm, fragment ion tolerance of 0.6 mass unit. The search allowed for variable oxidation of Met, phosphorylation of Ser, Thr, and Tyr, and carbamidomethylation of Cys. Each sample was run twice (*n* = 2).
