# Supplementary material for: Data on peptides identified by mass spectrometry analysis of in vitro DYRK1A-mediated phosphorylation sites on GLI1
Source: Data Brief. 2017 Oct 2;15:577–83. doi: 10.1016/j.dib.2017.09.057 (PMC5651491; doi:10.1016/j.dib.2017.09.057)
Supplement: Supplementary file 1 — Supplementary material [file mmc1.docx]

Conflicts of interest:

none
